# Feature selection with the R package *MXM*

**DOI:** 10.12688/f1000research.16216.2

**Published:** 2019-09-30

**Authors:** Michail Tsagris, Ioannis Tsamardinos

**Affiliations:** 1 Department of Economics, University of Crete, Rethymnon, 74100, Greece; 2 Department of Computer Science, University of Crete, Heraklion, Crete, 70013, Greece; 3 Statistical Learning Lab, Foundation of Research and Technology Hellas, Heraklion, Crete, 70013, Greece; 4 Institute of Applied and Computational Mathematics, Foundation of Research and Technology Hellas, Heraklion, Crete, 70013, Greece; 5 Gnosis Data Analysis (PC), Heraklion, Crete, 71305, Greece

**Keywords:** Feature selection, algorithms, R package, computational efficiency

## Abstract

Feature (or variable) selection is the process of identifying the minimal set of features with the highest predictive performance on the target variable of interest. Numerous feature selection algorithms have been developed over the years, but only few have been implemented in R and made publicly available R as packages while offering few options. The R package *MXM* offers a variety of feature selection algorithms, and has unique features that make it advantageous over its competitors: a) it contains feature selection algorithms that can treat numerous types of target variables, including continuous, percentages, time to event (survival), binary, nominal, ordinal, clustered, counts, left censored, etc; b) it contains a variety of regression models that can be plugged into the feature selection algorithms (for example with time to event data the user can choose among Cox, Weibull, log logistic or exponential regression); c) it includes an algorithm for detecting multiple solutions (many sets of statistically equivalent features, plain speaking, two features can carry statistically equivalent information when substituting one with the other does not effect the inference or the conclusions); and d) it includes memory efficient algorithms for high volume data, data that cannot be loaded into R (In a 16GB RAM terminal for example, R cannot directly load data of 16GB size. By utilizing the proper package, we load the data and then perform feature selection.). In this paper, we qualitatively compare *MXM* with other relevant feature selection packages and discuss its advantages and disadvantages. Further, we provide a demonstration of *MXM*’s algorithms using real high-dimensional data from various applications.

## Introduction

Given a target (response or dependent) variable *Y* of *n* measurements and a set **X** of *p* features (predictor or independent variables) the problem of feature (or variable) selection (FS) is to identify the minimal set of features with the highest predictability ^a^ on the target variable (outcome) of interest. The natural question that arises, is why should researchers and practitioners perform FS. The answer to this is for a variety of reasons ^1^, such as: a) many features may be expensive (and/or unnecessary) to measure, especially in the clinical and medical domains; b) FS may result in more accurate models (of higher predictability) by removing noise while treating the curse-of-dimensionality; c) the final produced parsimonious models are computationally cheaper and often easier to understand and interpret; d) future experiments can benefit from prior feature selection tasks and provide more insight into the problem of interest, its characteristics and structure. e) FS is indissolubly connected with causal inference that tries to identify the system’s causal mechanism that generated the data.

R contains thousands of packages, but only a small portion of them are dedicated to the task of FS, yet offering limited or narrow capabilities. For example, some packages accept few or specific types of target variables (e.g. binary and multi-class only). This leaves many types of target variables, e.g. percentages, left censored, positive valued, matched case-control data, etc., untreated. The availability of regression models for some types of data is rather small. Count data is such an example, for which Poisson regression is the only model considered in nearly all R packages. Most algorithms including statistical tests offer limited statistical tests, e.g. likelihood ratio test only. Almost all available FS algorithms are devised for large sample sized data, thus they cannot be used in many biological settings where the number of observations rarely (or never in some cases) exceeds 100, but the number of features is in the order of tens of thousands. Finally, some packages are designed for high volume data ^b^ only.

In this paper we present *MXM* ^c^ ^2^; an R package that overcomes the above shortcomings. It contains many FS algorithms ^d^, which can handle numerous and diverse types of target variables, while offering a pool of regression models to choose from and feed the FS algorithms. There is a plethora of statistical tests (likelihood-ratio, Wald, permutation based) and information criteria (BIC and eBIC) to plug into the FS algorithms. *MXM* offers algorithms that work with small and large sample sized data, algorithms that have been customized for high volume data, and an algorithm that returns multiple sets of statistically equivalent features are some of the key characteristics of *MXM*.

Over the next sections, a brief qualitative comparison of *MXM* with other packages available on CRAN and Bioconductor is presented, its (dis)advantages are discussed, its FS algorithms and related functions are mentioned. Finally a demonstration takes place, applying some FS algorithms available in *MXM* on real high dimensional data.

## The R package *MXM*

### *MXM* versus other R packages

**Figure 1.**
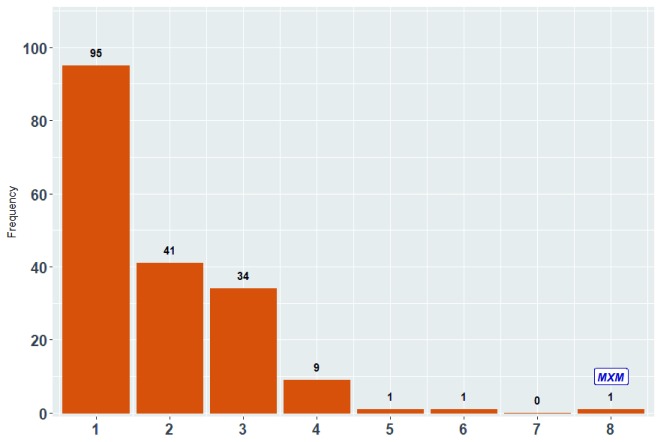
Frequency of FS related R packages handling different types of target variables. The horizontal axis shows the number of types (any combinations) of target variables from Table 1. For example, there 95 R packages that can handle only 1 type (any type) of target variable, 41 packages that can handle any 2 types of target variables, while *MXM* is the only one that handles all of them.

When searching for FS packages on CRAN and Bioconductor repositories using the keywords "feature selection", "variable selection", "selection", "screening" and "LASSO" ^e^, we detected 184 R packages until the 7th of May 2018 ^f^. Table 1 shows the frequency of the target variable types those packages accept ^g^, while Figure 1 shows the frequency of R packages whose FS algorithms can treat at least *k* types of target variables, for *k* = 1, 2, . . . , 8, of those presented in Table 1. Table 2 presents the frequency of pairwise types of target variables offered in R packages and Table 3 contains information on packages allowing for less frequent regression models. Most packages offer FS algorithms that are oriented towards specific types of target variables, methodology and regression models, offering at most 3–4 options. Out of these 184 packages, 65 (35.32%) offer LASSO type FS algorithms, while 19 (10.32%) address the problem of FS from the Bayesian perspective. Only 2 (1.08%) R packages treat the case of FS with multiple datasets ^h^ while only 4 (2.17%) packages are devised for high volume data.

**Table 1.** Frequency of CRAN and Bioconductor FS related packages in terms of the target variable they accept. The percentage-wise number (out of 184) appears inside the parentheses.

Target type	Binary	Nominal	Continuous	Counts
Frequency (%)	107 (58.15%)	31 (16.85%)	120 (65.22%)	29 (15.76%)
Target type	Survival	Case-control	Ordinal	Multivariate
Frequency (%)	27 (14.67%)	3 (1.63%)	3 (1.63%)	11 (5.97%)

**Table 2.** Cross-tabulation of the FS packages in R based on the target variable. There are 108 packages which handle binary target variables, 59 packages offering algorithms for binary and continuous target variables and only one package handling ordinal and nominal target variables, etc.

	Binary	Nominal	Continuous	Counts	Survival	Case-control	Ordinal	Multivariate
Binary	*108*							
Nominal	*32*	32						
Continuous	*59*	13	120					
Counts	28	3	28	29				
Survival	18	5	17	7	27			
Case-control	1	1	1	1	1	3		
Ordinal	4	1	2	2	1	1	4	
Multivariate continuous	4	3	8	4	3	1	1	11

**Table 3.** Frequency of other types of regression models for FS treated by R packages on CRAN and Bioconductor. The percentage-wise number appears inside the parentheses.

Regression models	Robust	GLMM	GEE	Functional
Frequency (%)	4 (2.19%)	8 (4.37%)	2 (1.09%)	2 (1.09%)

Table 4 summarizes the types of target variables treated by *MXM*’ FS algorithms along with the appropriate regression models that can be employed. The list is not exhaustive, as in some cases the type of the predictor variables (continuous or categorical) affects the decision of using a regression model or a test (Pearson and Spearman for continuous and *G* ^2^ test of independence for categorical). With percentages for example, *MXM* offers numerous regression models to plug into its FS algorithms: beta regression, quasi binomial regression or any linear regression model (robust or not) after transforming the percentages using the logistic transformation. For repeated measurements (correlated data), there are two options offered, the GeneralisedGeneralised Linear Mixed Models (GLMM) and Generalised Estimating Equations (GEE) which can also be used with various types of target variables, not mentioned here. We emphasize that *MXM* is the only package that covers all types of response variables mentioned on Table 1, many types of which are not available in any other FS package, such as left censored data for example. *MXM* also covers 3 out 4 cases that appear on Table 3.

**Table 4.** A brief overview of the types of target variables and regression models in *MXM*.

Target variable type	Regression model or test
Continuous and percentages without zeros	Linear, MM and quantile regression, Pearson and Spearman correlation coefficients
Multivariate continuous	Multivariate linear regression
Compositional data	Multivariate linear regression
(Strictly) positive valued	Gaussian and Gamma regression with a log-link
Percentages with or without zeros	Beta regression and quasi binomial regression
Counts	Poisson, quasi Poisson, negative binomial and zero inflated Poisson regression
Binary	Logistic regression, quasi binomial regression and *G* ^2^ test of independence
Nominal	Multinomial regression and *G* ^2^ test of independence
Ordinal	Ordinal regression
Number of successes out of trials	Binomial regression
Time-to-event	Cox, Weibull and exponential regression
Matched case-control	Conditional logistic regression
Left censored	Tobit regression
Repeated/clustered, longitudinal	Generalised linear mixed models (GLMM) and Generalised estimating equations (GEE)

### The *MXM*’s FS algorithms and comparison with other FS algorithms

Most of the currently available FS algorithms in the *MXM* package have been developed by the creators and authors of the package (see the last column of Table 5). The Incremental Association Markov Blanket (IAMB) algorithm was suggested by 3. The algorithm first performs the classical Forward Selection Regression (FSR) and then performs a variant of the classical Backward Selection Regression (BSR). Instead of removing the least significant feature detected at each step, it removes all non significant features. The Max Min Parents and Children (MMPC) algorithm also developed ^4^, is designed for small sample sized data and also performs a variant of FSR. At every step, when searching for the next best feature it does not use all currently selected features, but subsets of them and the non significant features are removed from further consideration. The Max Min Markov Blanket (MMMB) algorithm proposed by 4 picks the features MMPC selected and applies MMPC using each of them as target variable. The Statistically Equivalent Signatures (SES) algorithm suggested by 2 builds upon MMPC in order to identify statistically equivalent features; features that carry the same information with a selected feature. The Forward Backward with Early Dropping (FBED) algorithm is the most recently suggested FS algorithm by 5 (two authors of the *MXM* R package) and improves the computational cost of FSR by removing the non significant features at every step. In the end all the removed features can be tested again multiple times. Finally BSR is applied to remove possibly falsely selected features. Finally, the generalised Orthogonal Matching Pursuit (gOMP) ^6^ is a generalisation of OMP (Orthogonal Matching Pursuit) ^7– 9^. OMP applies a residual based FSR with continuous target variables only. We have generalised it to accept numerous types of target variables, such as binary, nominal, ordinal, counts, multivariate, time-to-event, left censored, proportions, etc, while for each of them the user can choose their regression model to employ.

**Table 5.** Algorithm suggestion according to combinations of sample size (n) and number of features (p), multiple solutions and high volume data.

Algorithm	Cases						
	n small & p small	n small & p big	n big & p small	n big & p big	High volume data	Multiple solutions	Authors development
BSR			✓				
FBED			✓	✓	✓		✓
FSR			✓				
gOMP			✓	✓	✓		✓
IAMB			✓				✓
MMMB	✓	✓	✓				✓
MMPC	✓	✓	✓				✓
SES	✓	✓	✓			✓	✓

These algorithms have been tested and compared with other state-of-the-art algorithms under different scenarios and types of data. IAMB ^3^ was on par with or outperforming competing machine learning algorithms, when both the target variable and features are categorical. MMPC and MMMB algorithms ^4^ were tested in the context of Bayesian Network learning showing great success with MMPC shown to achieve excellent false positive rates ^10^. MMPC formed the basis of Max Min Hill Climbing (MMHC) ^11^, a prototypical algorithm for learning the structure of a Bayesian network which outperformed all other Bayesian network learning algorithms with categorical data. For time-to-event and nominal categorical target variable, MMPC ^12^, and 13 respectively, outperformed or was on par with LASSO and other FS algorithms. SES ^2^ was contrasted against LASSO with continuous, binary and survival target variables, resulting in similar conclusions as before. With temporal and time-course data, SES ^14^ outperformed the LASSO algorithm ^15^ both in predictive performance and computational efficiency. FBED ^5^ was compared to LASSO for the task of binary classification with sparse data exhibiting performance similar to that of LASSO. As for gOMP, our experiments have showed very promising results, achieving similar or better performance, while enjoying higher computational efficiency than LASSO ^6^.

### Advantages and disadvantages of *MXM*’s FS algorithms

The main advantage of *MXM* is that all FS algorithms accept numerous and diverse types of target variables. MMPC, SES and FBED treat all types of target variables presented in Table 4, while gOMP handles fewer types ^i^.

*MXM* is the only R package that offers many different regression models to be employed by the FS algorithms, even for the same type of response variable, such as Poisson, quasi Poisson, negative binomial and zero inflated Poisson regression for count data. For repeated measurements, the user has the option of using GLMM or the GEE methodology (the latter with more options in the correlation structure) and for time-to-event data, Cox, Weibull and exponential regression models are the available options.

A range of statistical tests and methodologies to select the features is offered. Instead of the usual log-likelihood ratio test, the user has the option to use the Wald test or produce a p-value based on permutations. The latter is useful and advised when the sample size is small, emphasizing the need for use of MMPC and SES, both of which are designed for small sample sized datasets. FBED on the other hand, appart from the log-likelihood ratio test offers the possibility of using information criteria, such as BIC ^16^ and eBIC ^17^.

No p-values correction (e.g. Benjamini and Hochberg ^18^,) is applied. Specifically MMPC (and SES essentially) has been proved to control the False Discovery Rate ^19^. However, we allow for permutation-based p-values when performing MMPC and SES. FBED addresses this issue either by removing the non-significant variables or by using information criteria such as the extended BIC ^17^. Borboudakis and Tsamardinos ^5^ have conducted experiments showing that FBED reduces the percentage of falsely selected features. gOMP on the other hand relies on correlations and does not select the candidate feature using p-values.

Statistically Equivalent Signatures (SES) ^2, 20^ builds upon the ideas of MMPC and returns multiple (statistically equivalent) sets of predictor variables, making it one of the few FS algorithms suggested in the literature, and available in CRAN, with this trait ^21^. demonstrated that multiple, equivalent prognostic signatures for breast cancer can be extracted just by analyzing the same dataset with a different partition in training and test sets, showing the existence of several genes which are practically interchangeable in terms of predictive power. SES along with MMPC are two among the few algorithms, available on CRAN , that can be used to perform FS with multiple datasets in a meta-analytic way, following ^22^. *MXM* contains FS algorithms for small sample sized data (MMPC, MMMB, and SES) ^j^ and for large sample sized data (FBED, gOMP). FBED and gOMP have been adopted for high volume data, going beyond the limits of R. The importance of these customizations can be appreciated by the fact that nowadays large scale datasets are more frequent than before. Since classical FS algorithms cannot handle such data, modifications must be made, in an algorithm level, in a memory efficient manner, in a computer architecture level, and/or in any other way. *MXM* is using the efficient memory handling R package *bigmemory* ^23^.

Finally, many utility functions are available, such as constructing a model from the object an algorithm returned, construct a regression model, long verbose output with useful information, etc. Using *hash* objects, the computational cost of MMPC and SES is significantly reduced. Further, communication between the input and outputs of the algorithms is possible. The univariate associations computed by MMPC can be supplied to SES and FBED, and vice versa, and save computational time.

Not all *MXM*’s FS algorithms and all regression models used are computationally efficient. The (algorithmic) order of complexity of the FS algortihms is comparable to state-of-art FS algorithms, but the nature of the other algorithms is such that many regression models must be fit increasing the computational burden. In addition, R itself does not allow for further speed improvement. For example, MMPC can be slow for many regression models, such as negative binomial or ordinal regression for which we rely on implementations in other R packages. gOMP, on the other hand, is the most efficient algorithm available in *MXM* ^k^ gOMP superseded the LASSO implementation in the package *glmnet* ^24^ in both time and performance., because it is residual based and few regression models are fit. With clustered/longitudinal data, SES (and MMPC) were shown to scale to tens of thousands and be dramatically faster than LASSO ^14^. Computational efficiency is also programming language-dependent. Most of the algorithms are currently written in R and we are constantly working towards transferring them to C++ so as to decrease the computational cost significantly.

It is impossible to cover all cases of target variables; we have no algorithms for time series, and do not treat multi-state time-to-event target variables for example, yet we regularly search for R packages that treat other types of target variables and link them to *MXM* ^l^. All algorithms are limited to linear or generalised linear relationships, and we plan to address this issue in the future. The gOMP algorithm does not accept all types of target variables and works only with continuous predictor variables. This is a limitation of the algorithm, but we plan to address this in the future as well.

Cross-validation functions currently exist only for MMPC, SES and gOMP, but performance metrics are not available for all target variables. When the target variable is binary AUC, accuracy or the F score can be utilised, when the target takes continuous values, the mean squared error, the mean absolute error or the proportion of variance explained can be used, whereas with survival target variables the concordance index can be computed. Left censored data, is an example of target variable whose predictive performance estimation is not offered. A last drawback is that currently *MXM* does not offer graphical visualization of the algorithms and of the final produced models.

### Which FS algorithm from *MXM* to use and when

In terms of sample size, FBED and gOMP are generally advised for large-sample-sized datasets, whereas MMPC and SES are designed mainly for small-sample-sized datasets ^m^. In the case of a large sample size and few features, FSR or BSR are also suggested. In terms of number of features, gOMP is the only algorithm that scales up when the number of features is in the order of the hundreds of thousands. gOMP is also suitable for high volume data that contain a high number of features, really large sample sizes or both. FBED has been customized to handle high volume data as well, but with large sample sizes and only a few thousand features. If the user is interested in discovering more than one set of features, SES is suitable for returning multiple solutions, which are statistically equivalent. With multiple datasets ^n^, both MMPC and SES are currently the only two algorithms that can handle some cases (both the target variable and the set of features are continuous). As for the availability of the target variable, MMPC, SES and FBED handle all types of target variables available in *MXM*, listed in Table 4, while gOMP accepts fewer types of target variables. Regarding the type of features, gOMP currently works with continuous features only, whereas all other algorithms accept both continuous and categorical features. All this information is presented in Table 5.

## Methods

### Implementation

*MXM* is an R package that makes use of (depends or imports) other packages that offer regression models or utility functions.

- bigmemory: for large volume data.
- doParallel: for parallel computations.
- coxme: for frailty models.
- geepack: for GEE models.
- lme4: for mixed models.
- MASS: for negative binomial regression, ordinal regression and robust (MM type) regression.
- nnet: for multinomial regression.
- ordinal: for ordinal regression.
- quantreg: for quantile regression.
- *stats* (built-in package): for generalised linear models.
- survival: for survival regression and Tobit regression.
- Rfast: for computational efficiency.

### FS-related functions and computational efficiency tricks

*MXM* contains functions for returning the selected features for a range of hyper-parameters for each algorithm. For example, **mmpc.path** runs MMPC for multiple combinations of *threshold* and *max _k_*, and **gomp.path** runs **gOMP** for a range of stopping values. The exception is with FBED, for which the user can give a vector of values of *K* in **fbed.reg** instead of a single value. Unfortunately, the path of significance levels cannot be determined at a single run ^o^.

MMPC and SES have been implemented in such a way that the user has the option to store the results (p-values and test statistic values) from a single run in a *hash* object. In subsequent runs, with different hyper-parameters this can lead to significant amounts of computational savings because it avoids performing tests that have been already been performed. These two algorithms give the user an extra advantage. They can search for the subset of feature(s) that rendered one more specific feature(s) independent of the target variable by using the function **certificate.of.exclusion**.

FBED, SES and MMPC are three algorithms that share common grounds. The list with the results of the univariate associations (test statistic and logged p-value) can be calculated from either algorithm and be passed onto any of them. When one is interested in running many algorithms, this can reduce the computational cost significantly. Note also that the univariate associations in MMPC and SES can be calculated in parallel, with multi-core machines. More FS related functions can be found in *MXM*’s reference manual and vignettes section available on CRAN.

### Operation

*MXM* is distributed as part of the CRAN R package repository and is compatible with Mac OS X, Windows, Solaris and Linux operating systems. Once the package is installed and loaded


> install.packages("MXM")
> library(MXM)


it is ready to be used without internet connection. The system requirements are documented on *MXM*’s webpage on CRAN.

## Use cases

We will now demonstrate some FS algorithms available in *MXM*, using real datasets. Specifically we will show the relevant commands and describe part of their output. With user-friendliness taken into consideration, extra attention has been put in keeping the functions within the MXM package as consistent as the nature of the algorithms allows for, in terms of syntax, required input objects and parameter arguments. Table 6 contains a list of the current FS algorithms, but we will demonstrate some of them here. In all cases, the arguments "target", "dataset" and "test" refer to the target variable, set of features and type of regression model to be used.

**Table 6.** An overview of the main FS algorithms in *MXM*.

R Function	Algorithm
MMPC	Max-Min Parents and Children (MMPC)
SES	Statistically Equivalent Signatures (SES)
mmmb	Max-Min Markov Blanket (MMMB)
fs.reg	Forward selection (FSR)
bs.reg	Backward selection (BSR)
iamb	Incremental Association Markov Blanket (IAMB)
fbed.reg	Forward-Backward with Early Dropping (FBED)
gomp	Generalized Orthogonal Matching Pursuit (gOMP)

We will use a variety of target variables and in some examples, we will show the results produced with different regression models. Nearly all datasets (except for the first one) contain tens of thousands of features. The difference is with the target variable or interest. For example, when trying to select features for a time-to-event target variable survival regression should be employed, with continuous target variable, linear regression can be employed, whereas for counts, negative binomial or quasi Poisson regression could be employed by the FS algorithm. Under no circumstances should the following examples be considered experimental or for the purpose of comparison. They are only for the purpose of algorithms’ demonstration, to give examples of different types of target variables and to show how the algorithms work. All computations took place in a desktop computer with Intel Core i5-4690K CPU @3.50GHz and 32 GB RAM.

### Survival (or time-to-event) target variable

The first dataset we used concerns breast cancer, with 295 women selected from the fresh-frozen–tissue bank of the Netherlands Cancer Institute ^25^. The dataset contains 70 features and the target variable is time to event, with 63 censored values ^p^. We need this information, to be passed as a numerical variable indicating the status (0 = censored, 1 = not censored), for example (1, 1, 0, 1, 1, 1, . . . ). We will make use of the R package survival ^26^ for running the appropriate models (Cox and Weibull regression) and show the FBED algorithm with the default arguments. Part of the output is presented below. Information on the selected features, their test statistic and their associated logarithmically transformed p-value ^q^, along with some information on the number of regression models fitted is displayed.


> target <- survival::Surv(y, status)
> MXM::fbed.reg(target = target, dataset = dataset, test = "censIndCR")

$res
  sel     stat      pval
1  28 8.183389 -5.466128
2   6 5.527486 -3.978164

$info
    Number of vars Number of tests
K=0              2              73


The first column of $res denotes the selected features, i.e. the 28th and the 6th feature were selected. The second and third columns refer to the feature(s)’s associated test statistic and p-value. The $info informs the user on the value of K used, the number of selected features and the number of tests (or regression models) performed.

The above output was produced using Cox regression. If we used Weibull regression instead ( *test = "testIndWR"*), the output would be slightly different. Only one feature (the 28th) was selected, and FBED performed 75 tests (based upon 75 fitted regression models).


> MXM::fbed.reg(target = target, dataset = dataset, test = "censIndWR")

$res
     sel     stat      pval
Vars  28 8.489623 -5.634692

$info
    Number of vars Number of tests
K=0              1              75


### Unmatched case control target variable

The second dataset we used again concerns breast cancer ^27^ and contains 285 samples over 17,187 gene expressions (features). Since the target variable is binary, logistic regression was employed by gOMP.


> MXM::gomp(target = target, dataset = dataset, test = "testIndLogistic")


The element *res* presented below is one of the elements of the returned output. The first column shows the selected variables in order of inclusion and the second column is the deviance of each regression model. The first line refers to the regression model with 0 predictor variables (constant term only).


$res
      Selected Vars  Deviance
 [1,]             0  332.55696
 [2,]          4509  156.33519
 [3,]         17606  131.04428
 [4,]          3856  113.78382
 [5,]         10101   95.76704
 [6,]         16759   80.25748
 [7,]          6466   67.78120
 [8,]         11524   54.54652
 [9,]          9794   44.17957
[10,]          4728   36.52319
[11,]          3620   20.48441
[12,]         13127   5.583645e-10


### Longitudinal data

The next dataset we will use is NCBI Gene Expression Omnibus accession number GSE9105 ^28^, which contains 22,283 features about skeletal muscles from 12 normal, healthy glucose-tolerant individuals exposed to acute physiological hyperinsulinemia, measured at 3 distinct time points. Following ^14^, we will also use SES and not FBED because the sample size is small. The grouping variable that identifies the subject along with the time points is necessary in our case. If the data were clustered data, i.e. families, where no time is involved, the argument "reps" would not be provided. The user has the option to use GLMM ^29^ or GEE ^30^. The output of SES (and of MMPC) is long and verbose, and thus we present the first 10 set of equivalent signatures. The first row is the set of selected features, and every other row is an equivalent set. In this example, the last four columns are the same and only the first changes. This means, that the feature 2683 has 9 statistically equivalent features, (2, 7, 10, ...).


> MXM::SES.temporal(target = target, reps = reps, group = group,
                         dataset = dataset, test = "testIndGLMMReg")
@signatures[1:10,]
      Var1 Var2 Var3  Var4  Var5
 [1,] 2683 6155 9414 13997 21258
 [2,]    2 6155 9414 13997 21258
 [3,]    7 6155 9414 13997 21258
 [4,]   10 6155 9414 13997 21258
 [5,]   18 6155 9414 13997 21258
 [6,]  213 6155 9414 13997 21258
 [7,]  393 6155 9414 13997 21258
 [8,]  699 6155 9414 13997 21258
 [9,]  836 6155 9414 13997 21258
[10,] 1117 6155 9414 13997 21258


### Continuous target variable

The next dataset we consider is from Human cerebral organoids recapitulate gene expression programs of fetal neocortex development ^31^. The data are pre-processed RNA-seq, thus continuous data, with 729 samples and 58, 037 features. We selected the first feature as the target variable and all the rest were considered to be the features. In this case we used FBED and gOMP, employing the Pearson correlation coefficient because all measurements are continuous.

FBED performed 123, 173 tests and selected 63 features.


> MXM::fbed.reg(target = target, dataset = dataset, test = "testIndFisher")

$info
    Number of vars Number of tests
K=0             63          123173


gOMP on the other hand was more parsimonious, selecting only 8 features. At this point we must highlight the fact that the selection of a feature was based on the adjusted *R* ^2^ value. If the increase in the adjusted *R* ^2^ due to the candidate feature was more than 0.01 or (1/%), the feature was selected.


> MXM::gomp(target = target, dataset = dataset, test = "testIndFisher",
method = "ar2", tol = 0.01)

$res
       Vars adjusted R2
 [1,]     0   0.0000000
 [2,] 11394   0.3056431
 [3,]  4143   0.4493530
 [4,] 49524   0.4744709
 [5,]     8   0.4936872
 [6,] 29308   0.5096887
 [7,]  8619   0.5287238
 [8,]  3194   0.5411237
 [9,]  5958   0.5513510


### Count data

The final example is on discrete valued target variable (count data) for which Poisson and quasi-Poisson regression models will be employed by the gOMP algorithm. The dataset with GEO accession number GSE47774 ^32^ contains RNA-seq data with 256 samples and 43,919 features. We selected the first feature to be the target variable and all the rest are the features.

We ran gOMP using Poisson ( *test="testIndPois"*) and quasi Poisson ( *test="testIndQPois"*) regression models, but we changed the stopping value to *tol=12*. Due to over-dispersion (variance > mean), quasi Poisson is more appropriate ^r^ than Poisson regression that assumes that the mean and the variance are equal. When Poisson was used, 107 features were selected; since the wrong model was used, many false positive features were included, while with the quasi Poisson regression only 10 features were selected.


> MXM::gomp(target = target, dataset = dataset, test = "testIndQPois",
tol = 12)

$res
      Selected Vars   Deviance
 [1,]             0 3821661.14
 [2,]          6391  145967.17
 [3,]         12844  129639.56
 [4,]         26883  113706.51
 [5,]         32680  108387.15
 [6,]         29370  102407.46
 [7,]          4274   96817.48
 [8,]         43570   91373.77
 [9,]         43294   86125.30
[10,]         31848   81659.51
[11,]         38299   77295.71


### Applications of SES and gOMP

The case of ordinal target variable (i.e. very low, low, high, very high) has been treated previously ^33^ for unrevealing interesting features measuring the user perceived quality of experience with YouTube video streaming applications and the Quality of Service (target variable) of the underlying network under different network conditions.

More recently ^34^, applied MMPC in order to identify the features that provide novel information about steady-state plasma glucose (SSPG, a measure of peripheral insulin resistance) and are thus most useful for prediction ^35^. used gOMP and identified the viscoelastic properties of the arterial tree as an important contributor to the circulating bubble production after a dive. Finally ^36^, applied SES and gOMP were applied in the field of fisheries for identifying the genetic SNP loci that are associated with certain phenotypes of the gilthead seabream (Sparus aurata). Measurements from multiple cultured seabream families were taken, and since the data were correlated and GLMM was applied. The study led to a catalogue of genetic markers that set the ground for understanding growth and other traits of interest in Gilthead seabream, in order to maximize the aquaculture yield.

## Summary

We presented the R package *MXM* and some of its feature selection algorithms. We discussed its advantages and disadvantages and compared it, at a high level, with other competing R packages. We then demonstrated, using real high-dimensional data with a diversity of types of target variables, four FS algorithms, including different regression models in some cases.

The package is constantly being updated with new functions and improvements being added and algorithms being transferred to C++ to decrease the computational cost. Computational efficiency was mentioned as one of *MXM*’ disadvantage which we are trying to address. However, computational efficiency is one aspect, and flexibility another. Towards flexibility we plan to add of more regression models, more functionalities, options and graphical visualizations.

## Data availability

- The first dataset we used (survival target variable) is available from Computational Cancer Biology.
- The second dataset we used (unmatched case control target variable) is available from GEO.
- The third dataset we used (longitudinal data) is available from GEO.
- The fourth dataset we used (continuous target variable) is available from GEO.
- The fifth dataset we used (count data) is available from GEO.

## Software availability

MXM is available from: https://cran.r-project.org/web/packages/MXM/index.html.

Source code is available from: https://github.com/mensxmachina/MXM-R-Package

Archived source code at time of publication: https://doi.org/10.5281/zenodo.3458013 ^37^

License: GPL-2.

## Footnotes

^a^Predictive performance metrics include AUC, accuracy, mean squared error, mean absolute error, concordance index, F score, proportion of variance explained, etc.

^b^In statistics and in the R packages the term "big data" is used to refer to such data. In the computer science terminology, big data are of much higher volume and require specific technology. For this reason we chose to use the term "high volume" instead of "big data".

^c^MXM stands for Mens eX Machina, meaning "mind from the machine".

^d^MXM is mainly FS oriented, but it offers (Bayesian) network learning algorithms (for causal inference) as well. In fact, many feature selection algorithms offered in *MXM* are Bayesian network inspired.

^e^LASSO is a renowned FS algorithm standing for Least Absolute Shrinkage and Selection Operator

^f^We highlight the fact that especially on hrefhttps://cran.r-project.org/CRAN, packages are uploaded at a super-linear rate. Bioconductor is more strict with the addition of new packages. The phenomenon of abandoned or not maintained packages for a long time is not at all unusual. Such an example is "biospear", removed from CRAN (archived) in the 30th of April 2018. On the other hand we manualy added in our list a package that performs FS without mentioning it in its title.

^g^We manually examined each package to identify the types of target variables it accepts and regression models it offers.

^h^Instead of having a target variable and a set of features, one can have two or more sets of target variables and features. The algorithm we have devised for this case uses simultaneously all target variables and sets of features

^i^For this long list of available target variables and regression models, expanding Table 4, see Guide on performing FS with the R package *MXM*.

^j^The Wald and log-likelihood ratio tests are asymptotic tests. In addition, the fitted regression models require large sample size. With small sample sizes, the fitted regression models must not contain many predictor variables in order not to estimate many parameters. To address these issues both MMPC and SES perform conditional independence tests using subsets of selected variables, thus reducing the number of estimated parameters.

^k^Based on our experiments ^6^.

^l^Currently, with little effort, one should be able to plug-in their own regression model into some of the algorithms. We plan to expand this possibility for all algorithms.

^m^To the best of our knowledge there are not many FS algorithms dealing with small sample sized data.

^n^Instead of having one dataset only to analyze one might have multiple datasets from different sources. We do not combine all datasets into a larger one, neither perform FS for each dataset separately. Each step of the MMPC and SES algorithms is performed simultaneously to all datasets.

^o^We plan to implement this more efficiently in the future

^p^Censoring occurs when partial information about some observations is available. It might be the case that some individuals will experience the event after completion of the study. Or when an individual is not part of the study for anymore, for a reason other than the occurrence of the event of interest. In a study about cancer, for example, some patients may die of another cause, e.g. another disease or car accident for example. The survival times of those patients has been recorded, but offer limited information.

^q^The logarithm of the p-values is computed and return in order to avoid small p-values (less than the machine epsilon 10 ^−16^) being rounded to 0. This is a crucial and key element of the algorithms because they rely on the correct ordering of the p-values.

^r^Negative binomial regression, *test="testIndNB"* is another alternative option.
