# Supplementary material for: Feature selection with the R package MXM
Source: F1000Res. 2019 Sep 30;7:1505. Originally published 2018 Sep 20. [Version 2] doi: 10.12688/f1000research.16216.2 (PMC6792475; doi:10.12688/f1000research.16216.2)
Supplement: Peer review history

# Peer review: Reviewer response for version 2

**DOI:** 10.5256/f1000research.22822.r54472

**Published:** 2019-10-14

**Authors:** Huitong Qiu

**Affiliations:** 1 Department of Biostatistics, Johns Hopkins University, Baltimore, MD, USA

Thanks to the authors for responding to my comments. The authors have addressed all my concerns.

I confirm that I have read this submission and believe that I have an appropriate level of expertise to confirm that it is of an acceptable scientific standard.

# Peer review: Reviewer response for version 2

**DOI:** 10.5256/f1000research.22822.r54471

**Published:** 2019-10-01

**Authors:** Thodoris Kypraios

**Affiliations:** 1 School of Mathematical Sciences, University of Nottingham, Nottingham, UK

I am satisfied with the authors' response to my comments and the paper now looks in better shape in conjunction with replying to Huitong's comments.

I confirm that I have read this submission and believe that I have an appropriate level of expertise to confirm that it is of an acceptable scientific standard.

# Peer review: Reviewer response for version 1

**DOI:** 10.5256/f1000research.17707.r42926

**Published:** 2019-01-28

**Authors:** Huitong Qiu

**Affiliations:** 1 Department of Biostatistics, Johns Hopkins University, Baltimore, MD, USA

The manuscript introduces a new R package, MXM, that offers a variety of feature selection algorithms in regression models. The new package presents relevant contribution to the toolbox of feature selection algorithms by covering more types of target variables than most existing packages, accommodating data with small or big sample sizes, and providing additional functionalities and utility features. The manuscript also demonstrates the usage of several functions in the package by analyzing real data.

 Regarding the presentation of the manuscript, I share the same concern with reviewer Thodoris that the acronyms in the paper come with poor explanation. Although several of the acronyms are explained in Table 4 and Table 6, most of appearances of these acronyms have no reference to these tables or explanations. This makes the paper unnecessarily hard to read.

 Secondly, I think the demonstration of the usage of the package could be more informative with more interpretations. For example, how can we interpret the p-values, adjusted R-squares, and deviance in the results of the model fittings? High level descriptions of the algorithms behind the demonstrated functions could also help the reader better understand the results.

 Below are comments to specific places in the manuscript:

- The 3rd paragraph in Introduction mentions that statistical tests (likelihood ratio, Wald, permutation based) can be plugged into the feature selection algorithms that work with small sample sized data. The aforementioned tests traditionally rely on large sample theory. I wonder what adjustments are needed to accommodate small sample sizes?
- Tables 1, 2, and Figure 1 shows statistics of target variable types supported by a number of R packages. What algorithm was used to identify the types of target variables accepted by an R package?
- What does “multiple datasets” mean as in “Only 2 (1.08%) R packages teat the case of FS with multiple datasets ...” (last sentence of Paragraph 1 on Page 5)?
- I find the last paragraph on Page 5 particularly uneasy to read due to the acronyms used without explanation. Some of these are later explained in Table 6 while a couple are not (e.g., MMHC). I think it would be helpful to make a comprehensive table explaining the algorithm acronyms used in this paper, and refer to the table here.
- In the last paragraph on Page 5, there are multiple places that compare algorithms in terms of “(predictive) performance”. I think it would be more informative to clarify what performance metrics we are looking at.
- In Paragraph 5 on Page 6, the manuscript states that “SES ... returns multiple (statistically equivalent) sets of predictor variables ...”. What does statistically equivalent mean?
- What does the package name, MXM, stand for?
- The second last paragraph on Page 6 is a bit confusing to me. The paragraph starts with pointing out computational efficiency as a disadvantage of MXM. However, this is followed by explaining why gOMP is efficient, and pointing out that SES and MMPC scales better and run faster than LASSO package. These seem like advantages in computational efficiency.
- In Paragraph 2 of “FS-related functions” section: for MMPC and SES, storing the results from one run and passing it to subsequent runs can lead to significant computational savings. Are there savings because the trained models serve as good starting points in the subsequent runs?
- On Page 9 in the output of MXM::fbed.reg, there are p-values associated with each selected variable. How can we interpret these p-values? It seems that these p-values are not adjusted for the extra degrees of freedom from feature selection.

Below are some minor suggestions/typo fixes:

- In Abstract – “The R package MXM is such an example, which offers ...”: It’s unclear to me what “such an example” refers to. It’s clearer to state “The R package MXM offers ...” directly.
- Second paragraph in Introduction: “For example, packages that accept few or specific types of target variables.” → “For example, some packages accept few or specific types of target variables.” (make it a complete sentence.)
- In “MXM versus other R packages” section: “... can treat at least one type of target variable, ...” → “... can treat at least k types of target variables, for k = 1, 2, ..., 8, ...”
- In the last paragraph on Page 5, does “BN” refer to Bayesian network (which also appears in the same paragraph)?
- In Paragraph 5 on Page 6 – “... making it one one of the few FS algorithms ...”: remove the extra “one”.
- In Paragraph 6 on Page6 – “MXM is using an efficient memory handling R package.”: please cite the package.
- Last Paragraph on Page 7: “generalised” → “generalized” to be consistent with other places in the paper.
- First paragraph in Section “Use cases”: the argument “test” refers to the type of regression model to be used. Why not name the argument something like “model type”?
- Some of the citations are not easily distinguishable from footnotes. e.g., citation 21 for Rfast on Page 8 and footnote 7 are both superscripts.
- Paragraph 2 on Page 11: “gOMP on the other has ...” → “gOMP on the other hand ...”.
- First paragraph on Page 12 – “YouTube video streaming applications applications”: duplicate “applications”.

I confirm that I have read this submission and believe that I have an appropriate level of expertise to confirm that it is of an acceptable scientific standard, however I have significant reservations, as outlined above.

# Response

**Published:** 2019-07-12

**Authors:** Michail Tsagris

**Affiliations:** University of Crete, Greece

We are grateful to the reviewers for their on-the-spot comments which we have addressed. 

The manuscript introduces a new R package, MXM, that offers a variety of feature selection algorithms in regression models. The new package presents relevant contribution to the toolbox of feature selection algorithms by covering more types of target variables than most existing packages, accommodating data with small or big sample sizes, and providing additional functionalities and utility features. The manuscript also demonstrates the usage of several functions in the package by analyzing real data.

- Comment: Regarding the presentation of the manuscript, I share the same concern with reviewer Thodoris that the acronyms in the paper come with poor explanation. Although several of the acronyms are explained in Table 4 and Table 6, most of appearances of these acronyms have no reference to these tables or explanations. This makes the paper unnecessarily hard to read.
- Reply: We have added the interpretation of the acronyms when they ﬁrst appear, at various places within the text.
- Comment: Secondly, I think the demonstration of the usage of the package could be more informative with more interpretations. For example, how can we interpret the p-values, adjusted R-squares, and deviance in the results of the model ﬁttings? High level descriptions of the algorithms behind the demonstrated functions could also help the reader better understand the results. 
- Reply: We thank the reviewer for this comment. We have added a small description of each algorithm does in Section “The MXM’s FS algorithms and comparison with other FS algorithms”. This was also highlighted by Prof Kypraios. Also, in Section “Advantages and disadvantages of MXM’s FS algorithms” we have added a small paragraph regarding the p-values produced by the algorithms.

Below are comments to speciﬁc places in the manuscript:

- Comment: The 3rd paragraph in Introduction mentions that statistical tests(likelihood ratio, Wald, permutation based) can be plugged into the feature selection algorithms that work with small sample sized data. The aforementioned tests traditionally rely on large sample theory. I wonder what adjustments are needed to accommodate small sample sizes?
- Reply: We have added a foot note in page 5 regarding this. There as on we added this information at this point was because we discuss the suitability of the algorithms based on the sample size. For example, we say the MMPC is more suitable for small sample sized data and explain why. FBED on the other hand is devised for large sample sizes.
- Comment: Tables 1, 2, and Figure 1 shows statistics of target variable types supported by a number of R packages. What algorithm was used to identify the types of target variables accepted by an R package?
- Reply: We did this manually. We searched on CRAN and went through all packages one by one. We did this process twice to make sure we did not omit any package. Bear in mind that this search was made a few months ago.
- Comment: What does “multiple datasets” mean as in “Only 2 (1.08%) R packages teat the case of FS with multiple datasets ...” (last sentence of Paragraph 1 on Page 5)?
- Reply: We added a footnote at this point explaining what we mean by multiple datasets and how we perform feature selection in this case. The 1.08% means that only 2 R packages (available on CRAN or Bioconductor) perform feature selection with multiple datasets.
- Comment: I ﬁnd the last paragraph on Page 5 particularly uneasy to read due to the acronyms used without explanation. Some of these are later explained in Table 6 while a couple are not (e.g., MMHC). I think it would be helpful to make a comprehensive table explaining the algorithm acronyms used in this paper, and refer to the table here.
- Reply: We thank the reviewer for this comment. We have explained all acronyms in various place, whenever the relevant algorithm is ﬁrst mentioned.
- Comment: In the last paragraph on Page 5, there are multiple places that compare algorithms in terms of “(predictive)performance”. I think it would be more informative to clarify what performance metrics we are looking at.
- Reply: We have added a couple a sentence mentioning some predictive performance metrics for some target variables.
- Comment: In Paragraph 5 on Page 6, the manuscript states that “SES ... returns multiple (statistically equivalent) sets of predictor variables ...”. What does statistically equivalent mean?
- Reply: This comment was raised by the other reviewer as well. In the Abstract we have added a sentence inside parentheses brieﬂy explain this. Also in Section “The MXM’s FS algorithms and comparison with other FS algorithms” we have added one sentence when mention the SES algorithm (this is in magenta colour). This comment was also made by the ﬁrst reviewer and we have answered this previously.
- Comment: What does the package name, MXM, stand for?
- Reply: We thank the reviewer for this comment. We added the meaning of this acronym in the 4rth footnote of page 2. It stands for Mens ex Machina.
- Comment: The second last paragraph on Page 6 is a bit confusing to me. The paragraph starts with pointing out computational efﬁciency as a disadvantage of MXM. However, this is followed by explaining why gOMP is efﬁcient, and pointing out that SES and MMPC scales better and run faster than LASSO package. These seem like advantages in computational efﬁciency.
- Reply: We thank the reviewer for this clariﬁcation. Indeed the message is diluted. We have reworded this point, towards the end of page 5.
- Comment: In Paragraph 2 of “FS-related functions” section: for MMPC and SES, storing the results from one run and passing it to subsequent runs can lead to signiﬁcant computational savings. Are there savings because the trained models serve as good starting points in the subsequent runs?
- Reply: We have added a few sentences clarifying this point. All p-values are stored to avoid implementing all tests again in subsequent runs.
- Comment: On Page 9 in the output of MXM::fbed.reg, there are p-values associated with each selected variable. How can we interpret these p-values? It seems that these p-values are not adjusted for the extra degrees of freedom from feature selection.
- Reply: We do recognize this problem and are aware that the distribution of the test statistics is not the assumed one [1] and their associated p-values can be small [2]. We have added a small discussion on the p-values at the bottom of page 4-top of page 5 regarding this. The p-values are not FDR corrected, but we refer to the papers that mention this issue as well. With MMPC for example, there is no need to apply FDR because the algorithm controls the FDR [3]. [4] discusses methods that address this issue and they conclude that the FBED algorithm is orthogonal to those methods and could be used in conjunction with them. Closing this issue we will mention that it is not easy to control FDR in the context of feature selection; it is an open problem. It is commonly accepted in the statistical literature, and not only, that this leads to regression coefﬁcients are overestimated. A solution would be to ﬁt a LASSO regularised model, for example, and tune the penalty algorithm via cross-validation. However, the problem of FDR is not totally addressed, but rather bypassed.

Below are some minor suggestions/typo ﬁxes:

- Comment: In Abstract – “The R package MXM is such an example, which offers ...”: It’s unclear to me what “such an example” refers to. It’s clearer to state “The R package MXM offers ...” directly.
- Reply: We thank the reviewer for this suggestion. We have reworded this sentence.
- Comment: Second paragraph in Introduction: “For example, packages that accept few or speciﬁc types of target variables.” -> “For example, some packages accept few or speciﬁc types of target variables.” (make it a complete sentence.)
- Reply: We thank the reviewer for this syntactical mistake which we have now corrected.
- Comment: In “MXM versus other R packages” section: “... can treat at least one type of target variable, ...” -> “... can treat at least k types of target variables, for k = 1, 2, ..., 8, ...”
- Reply: We have changed this sentence as requested.
- Comment: In the last paragraph on Page 5, does “BN” refer to Bayesian network (which also appears in the same paragraph)?
- Reply: We substituted the BN to Bayesian network.
- Comment: In Paragraph 5 on Page 6 – “... making it one one of the few FS algorithms ...”: remove the extra “one”.
- Reply: We have removed the extra “one”.
- Comment: In Paragraph 6 on Page6 – “MXM is using an efﬁcient memory handling R package.”: please cite the package.
- Reply: We have mentioned and cited the relevant package.
- Comment: Last Paragraph on Page 7: “generalised” -> “generalized” to be consistent with other places in the paper.
- Reply: We changed everything to “generalised”.
- Comment: First paragraph in Section “Use cases”: the argument “test” refers to the type of regression model to be used. Why not name the argument something like “model type”?
- Reply: We have reworded this part of the manuscript and added some more information about its target.
- Comment: Some of the citations are not easily distinguishable from footnotes. e.g., citation 21 for Rfast on Page 8 and footnote 7 are both superscripts.
- Reply: We did not spot this issue in the paper. #
- Comment: Paragraph 2 on Page 11: “gOMP on the other has ...” -> “gOMP on the other hand ...”.
- Reply: We changed this as requested by the ﬁrst reviewer also.
- Comment: First paragraph on Page 12 – “YouTubevideostreamingapplicationsapplications”: duplicate “applications”.
- Reply: We removed the duplicate.

[1] Hastie Trevor, Tibshirani Robert and Friedman JH. The elements of statistical learning: data mining, inference, and prediction. New York: Springer, 2009.

[2] Frank E Harrell Jr. Regression modeling strategies. Springer. 2017.

[3] Ioannis Tsamardinos and Laura E Brown.Bounding the False Discovery Rate in Local Bayesian Network Learning. In AAAI, pages 110-1105, 2008.

[4] Giorgos Borboudakis and Ioannis Tsamardinos. Forward-backward selection with early dropping. Journal of Machine Learning Research, 20(8):1–39, 2019.

# Peer review: Reviewer response for version 1

**DOI:** 10.5256/f1000research.17707.r38571

**Published:** 2018-10-08

**Authors:** Thodoris Kypraios

**Affiliations:** 1 School of Mathematical Sciences, University of Nottingham, Nottingham, UK

**Summary**

 The paper is concerned with the method of feature selection using the R package MXM. The package appears to be fairly versatile in the sense that it can handle a huge variety of types of data. It can be very useful for applied researchers and, at the very least, it is another tool in the toolbox for the applied statistician. 

 I believe that the paper it will be a useful addition in the literature. However, I found difficult to read/understand in places. For example, is the MXM a package that only includes methodology that has been developed by the authors or does it include methods that have been developed by other researchers too? Either way is fine, but it would be helpful to clarify.

 Another major issue for me is that the paper is flooded with acronyms that I believe most users will be unfamiliar with. It would improve the paper's presentation significantly, if there is some explanation (a couple of sentences per method would suffice) about each of them. 

 Below are some more specific points to consider: 

**Abstract**

-  It it not clear when one first reads what "b) it contains a variety of regression models to plug into the feature selection algorithms;" means.
- c), "equivalent" in what sense?
-  "it includes memory efficient algorithms for high volume data, data that cannot be loaded into R" -> "it includes memory efficient algorithms for high volume data that often cannot be loaded easily into R"? 

**Introduction**

- "and easier to understand and interpret;" -> "and often easier to understand and interpret;"
-  "small portion" -> "small proportion"? 
- It would be good to Table 1 to have the totals.
- "These algorithms have been tested and compared with other state-of-the-art algorithms under different scenarios and types of data." Any references?
- I find hard to read the "Comparisons of MXM’ FS algorithms with other FS algorithms" because there are a bunch of acronyms used that I (and presumably other readers) don't know what they mean; it would be good to say a few words about what each algorithm is doing.
-  "anecdotal" -> preliminary?
- It would be good to say what is g-OMP (as well as the other methods) doing?

 

**Methods**

- In the example "Survival (or time-to-event) target variable", a survival model is fitted and the MXM package is used and its output is presented, but I am unclear as to what is the objective (in terms of the data analysis) and what does the output mean.
- The above comments applies to the above datasets; it is crucial that reader knows what is the statistical objective first, and then to explain what the outcome of the package means. 
- page 11: "gOMP on the other" -> "gOMP on the other _hand_" ?

I confirm that I have read this submission and believe that I have an appropriate level of expertise to confirm that it is of an acceptable scientific standard, however I have significant reservations, as outlined above.

# Response

**Published:** 2019-07-12

**Authors:** Michail Tsagris

**Affiliations:** University of Crete, Greece

We are grateful to the reviewers for their on-the-spot comments.

The paper is concerned with the method of feature selection using the R package MXM. The package appears to be fairly versatile in the sense that it can handle a huge variety of types of data. It can be very useful for applied researchers and, at the very least, it is another tool in the toolbox for the applied statistician.

- Comment: I believe that the paper it will be a useful addition in the literature. However, I found difﬁcult to read/understand in places. For example, is the MXM a package that only includes methodology that has been developed by the authors or does it include methods that have been developed by other researchers too? Either way is ﬁne, but it would be helpful to clarify.
- Reply: We have added an extra column in Table 5 giving this information. Also, in Section “The MXM’s FS algorithms and comparison with other FS algorithms” where we describe the algorithms we have added the relevant references.
- Comment: Another major issue for me is that the paper is ﬂooded with acronyms that I believe most users will be unfamiliar with. It would improve the paper’s presentation signiﬁcantly, if there is some explanation (a couple of sentences per method would sufﬁce) about each of them.
- Reply: We have added the explanation of all acronyms when the algorithm is ﬁrst mentioned. Also in Section “The MXM’s FS algorithms and comparison with other FS algorithms” we brieﬂy mention how they work.

Below are some more speciﬁc points to consider:

Abstract

- Comment: It it not clear when one ﬁrst reads what "b)it contains a variety of regression models to plug into the feature selection algorithms;" means.
- Reply: We have added a sentence inside parentheses giving some examples of target variables and appropriate regression models explaining this sentence.
- Comment: c), "equivalent" in what sense? 
- Reply: We added a sentence inside parentheses explaining this sentence. Equivalent refers to the“information” a feature contains. If for example the information gain of a variable is not signiﬁcant it could be attributed to another feature that contains the same information as this one. Think for example collinear variables, such as length of left hand and length of the right hand. Either hand can be used in a regression model. This phenomenon is prevalent, but also not highly examined, in bioinformatics where the selected genes may not be the ones expected by the biologists, only because some equivalent genes were selected. Only few feature selection algorithms return all equivalent features and SES is one of them.
- Comment: "it includes memory efﬁcient algorithms for high volume data, data that cannot be loaded into R" -> "it includes memory efﬁcient algorithms for high volume data that often cannot be loaded easily into R"?
- Reply: We have added a sentence inside parentheses explaining this sentence. The term “big data” has wrongfully been used in many instances to denote large scale data. What we mean by high volume data is data whose size in Gb equals or exceeds the size of the available RAM in one’s computer and hence cannot be loaded into R.

Introduction

•Comment: "and easier to understand and interpret;" -> "and often easier to understand and interpret;" • Reply: We thank the reviewer for this grammatical suggestion. We changed it. • Comment: "small portion" -> "small proportion"?
- Reply: We did not change this word. We consider the word “portion” as a synonym for “share”. The word “proportion” is rather further away from our interpretation and the meaning of the word we wanted to use.
- Comment: It would be good to Table 1 to have the totals.
- Reply: We added the total (184 packages) in the caption of Table 1.
- Comment: "These algorithms have been tested and compared with other state-of-the-art algorithms under different scenarios and types of data." Any references?
- Reply: For each algorithm separately, we have added the relevant reference.
- Comment: I ﬁnd hard to read the "Comparisons of MXM’ FS algorithms with other FS algorithms" because there are a bunch of acronyms used that I (and presumably other readers) don’t know what they mean; it would be good to say a few words about what each algorithm is doing.
- Reply: We thank the reviewer for this comment. We have expanded this section by adding a short description for each algorithm.
- Comment: "anecdotal" -> preliminary?
- Reply: We changed the whole sentence and refer to the relevant paper that is a draft paper available on bioRxiv.
- Comment: It would be good to say what is g-OMP (as well as the other methods) doing?
- Reply: We have added a short description of what each feature selection algorithm does.

Methods

- Comment: In the example "Survival (or time-to-event) target variable", a survival model is ﬁtted and the MXM package is used and its output is presented, but I am unclear as to what is the objective (in terms of the data analysis) and what does the output mean.
- Reply: We thank the reviewer for this important comment. We have modiﬁed this example and the others. In addition, we have added a paragraph in the beginning of this section explaining the goal of this section.
- Comment: The above comments applies to the above datasets; it is crucial that reader knows what is the statistical objective ﬁrst, and then to explain what the outcome of the package means.
- Reply: Please see previous comment.
- Comment: page 11: "gOMP on the other" -> "gOMP on the other hand"?
- Reply: We added the word “hand”. 
